# The epidemiology of Hepatitis B, C and D in Germany: A scoping review

**DOI:** 10.1371/journal.pone.0229166

**Published:** 2020-03-09

**Authors:** Gyde Steffen, Ida Sperle, Siv Aina Leendertz, Navina Sarma, Sandra Beermann, Roma Thamm, Viviane Bremer, Ruth Zimmermann, Sandra Dudareva

**Affiliations:** 1 Department of Infectious Disease Epidemiology, Robert Koch Institute, Berlin, Germany; 2 Department of Infectious Disease Epidemiology, Translational Infrastructure Epidemiology of the German Centre for Infection Research, Robert Koch Institute, Berlin, Germany; 3 Department of Epidemiology and Health Monitoring, Robert Koch Institute Berlin, Berlin, Germany; 4 Centre for International Health Protection, Robert Koch Institute, Berlin, Germany; Centre de Recherche en Cancerologie de Lyon, FRANCE

## Abstract

**Background:**

Germany is considered to be a low prevalence country for viral Hepatitis B, C and D (HBV, HCV, HDV). However, the burden of disease can be high among subpopulations. To meet the world Health Organization (WHO) viral hepatitis (VH) elimination goals, a national strategy was developed by the German government in 2016. We performed a scoping review to understand the baseline epidemiological situation in Germany regarding burden of disease, sequelae and care of HBV, HCV and HDV as a reference to monitor the progress of the national VH elimination and to identify further knowledge gaps and research needs.

**Methods:**

The protocol of the systematic review was prepared following the PRISMA statement guidelines for scoping reviews. Relevant search terms were used to identify eligible studies according to the research questions. We searched six online databases for original work published between January 2005 and March 2017. Based on the identified references, a matrix was developed presenting the eligible literature by targeted population group and outcome category.

**Results:**

104 publications were eligible for extraction covering 299 outcome results. The population groups targeted in the identified studies included the general population and proxy populations, a range of clinical populations, people who inject drugs, men who have sex with men, healthcare workers, people in prisons and different migrant/mobile populations. Other vulnerable populations (e.g. sex workers) were not targeted. Overall, good evidence was found for HBV and HCV prevalence and HBV vaccination coverage in the GP and proxy populations. Evidence for these outcomes was weaker in populations at risk for VH. For HBV and HCV incidence and mortality, we identified large evidence gaps in all population groups. Outcomes on VH sequelae and care were mainly covered by studies in clinical populations of people living with viral hepatitis. For HDV the overall evidence available was scarce.

**Conclusions:**

We created a comprehensive evidence-based overview on the current epidemiological situation of viral hepatitis in Germany. We identified knowledge gaps for further research and established a baseline for future monitoring of viral hepatitis elimination goals in Germany.

## Background

Globally, viral hepatitis (VH) is a leading cause of morbidity and mortality. An estimated 257 and 71 million people are chronically infected with Hepatitis B (HBV) and Hepatitis C (HCV), respectively. Fifteen to twenty million (5.0% of those with HBV) are infected with Hepatitis D virus (HDV) [1]. In the World Health Organization (WHO) European Region, an estimated 15 and 14 million people are suffering from chronic HBV and HCV infection, respectively [2]. The major transmission routes of HBV, HCV and HDV are the exposure to infected blood, such as through unsafe injection practices, via sexual contact and transmission from mother to child during birth. As a result, some populations are more vulnerable to VH than others (hereafter populations at risk for VH) [3]. Chronic infections can cause liver cirrhosis and liver cancer and liver transplantation may be the ultima ratio for individuals with acute or chronic liver failure due to VH. According to estimates by the WHO, VH caused 1.34 million deaths globally in 2015 [4]. The more rare HDV leads to a more severe infection and the rapid progression to adverse outcomes.

In recent years, highly effective direct-acting antiviral treatment options for HCV infection have become available and this new treatment as well as the efficient HBV vaccine, available since 1982 in Germany, have made the elimination of VH a feasible target. In 2016, the first *Global Health Sector Strategy on VH 2016–2021 (GHSS)* [5] was endorsed by the World Health Assembly. It offers a strategy to work towards the elimination of VH as a major public health threat by 2030. In the same year, the *Action Plan for the Health Sector Response to VH in the World Health Organization (WHO) European Region* was developed and approved by the WHO Regional Committee [2].

Germany is considered a low-prevalence country for HBV and HCV, which was demonstrated in the last national population-based studies in adults and children (DEGS1, 2008–2011 and KiGGS Wave 1, 2009–2012) [6, 7]. However, the actual prevalence may be higher, as vulnerable populations at higher risk of VH infection are often less represented in population-based studies [8, 9].

In 2016, an integrated national strategy for HIV, HBV and HCV and other sexually transmitted diseases was approved by the German Government to improve the responses to these diseases [10]. Core points of this strategy are the extension of integrated prevention, testing and care services for reducing transmission, increasing early diagnosis and improving care with a focus on the further expansion of demand-orientated services for populations at risk for VH and with a high burden of VH and combatting stigma related to HIV, viral hepatitis and STI. To plan and implement new services as well as to control the success of the national strategy, differentiated knowledge about the epidemiological situation of HBV, HCV and HDV including data on burden of disease, sequelae and prevention/care in the general population (GP, refers to the national population) and populations at risk for VH in Germany is essential. Up to now, there has been no systematic overview of the existing scientific evidence regarding VH, which could serve as a reference for monitoring the progress of VH elimination. Therefore, we conducted a scoping review to provide an extensive baseline overview of the available evidence until 2017 regarding the extent, variety and nature of published literature in this area. Moreover, the aim of our study was to identify research gaps in order to support the planning of future research in the light of the hepatitis elimination goals [1].

## Methods

### Research question

Based on the aim of the scoping review, we formulated 13 specific research questions to identify relevant published literature covering several aspects of VH epidemiology in the GP and populations at risk for HBV, HCV and HDV in Germany:

1. What is the prevalence of HBV, HCV and HDV in Germany?
2. What is the incidence of HBV, HCV and HDV in Germany?
3. What is the mortality of HBV, HCV and HDV in Germany?
4. What are the genotypes of HBV, HCV and HDV?
5. What are the routes of transmission for HBV, HCV and HDV in Germany?
6. What is the proportion of HBV, HCV and HDV co-infections in Germany?
7. What is the burden of liver cirrhosis due to HBV, HCV and HDV in Germany?
8. What is the burden of hepatocellular carcinoma due to HBV, HCV and HDV in Germany?
9. What is the quality of life of people living with HBV, HCV and HDV?
10. What proportion of the population is diagnosed with HBV, HCV and HDV in Germany?
11. What proportion of the population is treated for HBV, HCV and HDV in Germany?
12. What is the proportion of liver transplantation due to HBV, HCV and HDV in Germany?
13. What is the HBV immunisation coverage in Germany?

HBV, HCV and HDV infection and HBV immunisation were defined as stated in the corresponding publications. This included infection/immunity confirmed by a serological test, self- reported or extracted from medical records. Liver cirrhosis, hepatocellular carcinoma and acute liver failure were defined as stated in the corresponding publication and included confirmation by clinical/serological tests, self- report or extraction from medical records. The proportion of HBV, HCV and HDV diagnosed individuals was defined as the proportion of positive tested individuals (as defined in the corresponding publication) not knowing resp. already knowing their infection status. The proportion of HBV, HCV and HDV treated individuals included intended, ongoing, finished and interrupted treatment as defined in the corresponding publication. The HBV immunisation coverage included complete and incomplete vaccination against HBV confirmed by a serological test, self-reported or extracted from a medical records as defined in the corresponding publication. Only descriptive epidemiology was considered, while analytical epidemiology (e.g. risk factors associated with outcomes in the research questions) was outside the scope of this review.

### Systematic literature search

The research and reporting methods of the scoping review were consistent with the *preferred reporting items for systematic reviews and meta-analysis extension for scoping reviews* (PRISMA-ScR) ([11], S1 Table) and partly with the Cochrane Collaboration (https://training.cochrane.org/handbook). An a priori protocol adapted from the protocol template of the *International prospective register of systematic reviews* (PROSPERO) [12] was prepared and will be provided on request from the corresponding author.

The literature search was conducted in six electronic databases (MEDLINE, EMBASE, Europe PMC, Scopus, Bielefeld Academic Search Engine (BASE) [13] and CC Med ([14], a database specialised in German medical journals). To develop a comprehensive search covering all eligible literature containing data on the epidemiology of HBV, HCV and HDV in Germany the 13 research questions were translated into a table of **P**opulations and **O**utcomes, adapted from the *PICO criteria* (**P**articipants, **I**nterventions, **C**omparator, **O**utcome [15]). Based on the table of outcomes three search strings were defined, one for each of the three viruses. The string also included appropriate regional terms limiting the search to data from Germany. The final search strings were checked for correctness by all team members. The search is attached as additional file (S1 Text). Before conducting the final search, internal and external experts indexed published journal articles relevant for each research question. To verify the search string, we screened if the index articles were represented in a test run of the search. The strings were run in the six electronic databases adapted to the format required for each database including medical subject headings for the search in PubMed (MeSH Terms) and EMBASE (EMTREE). The final search was conducted on 9^th^ March 2017 (cut-off date) and was restricted to references published between 1^st^ January 2005 to 9^th^ March 2017 in English or German language. The references that were retrieved through the online search were imported to Endnote and duplicates were removed. Additional relevant references were subsequently identified manually from the reference lists of the publications eligible forfulltext-screening. Moreover, reports of national surveillance data that are not published in the six electronic databases, were included in the review.

### Study selection criteria

We developed specific inclusion criteria to identify eligible publications. Included in the review were i) original works (analyses of data from observational studies (e.g. cross-sectional studies, cohort studies, case-control studies, case series from the national surveillance, of secondary or registry data and of reviews with a systematic approach), ii) Full-text-publications as peer-reviewed/non-peer-reviewed journal articles and other forms of publications (e.g. doctoral theses), iii) with end of data collection after 1^st^ January 205 and publication between 1^st^ January 2005 and 9^th^ March 2017 and iiii) published in German or English language. From cohort studies, only cross-sectional outcomes were considered and from case-control studies only data gained from cases. Publications without full text (e.g. conference abstracts) were excluded. Furthermore, publications that did not report basic demographics (e.g., age, sex) of participants or with major errors in reporting (e.g. error in numbers, wrong calculations) were excluded. If multiple publications reported identical outcomes from the same study or cohort without new aspects relevant for the research questions, the newer publication was excluded. For the specific study selection criteria see S1 Fig.

### Study selection process

**Fig 1:**
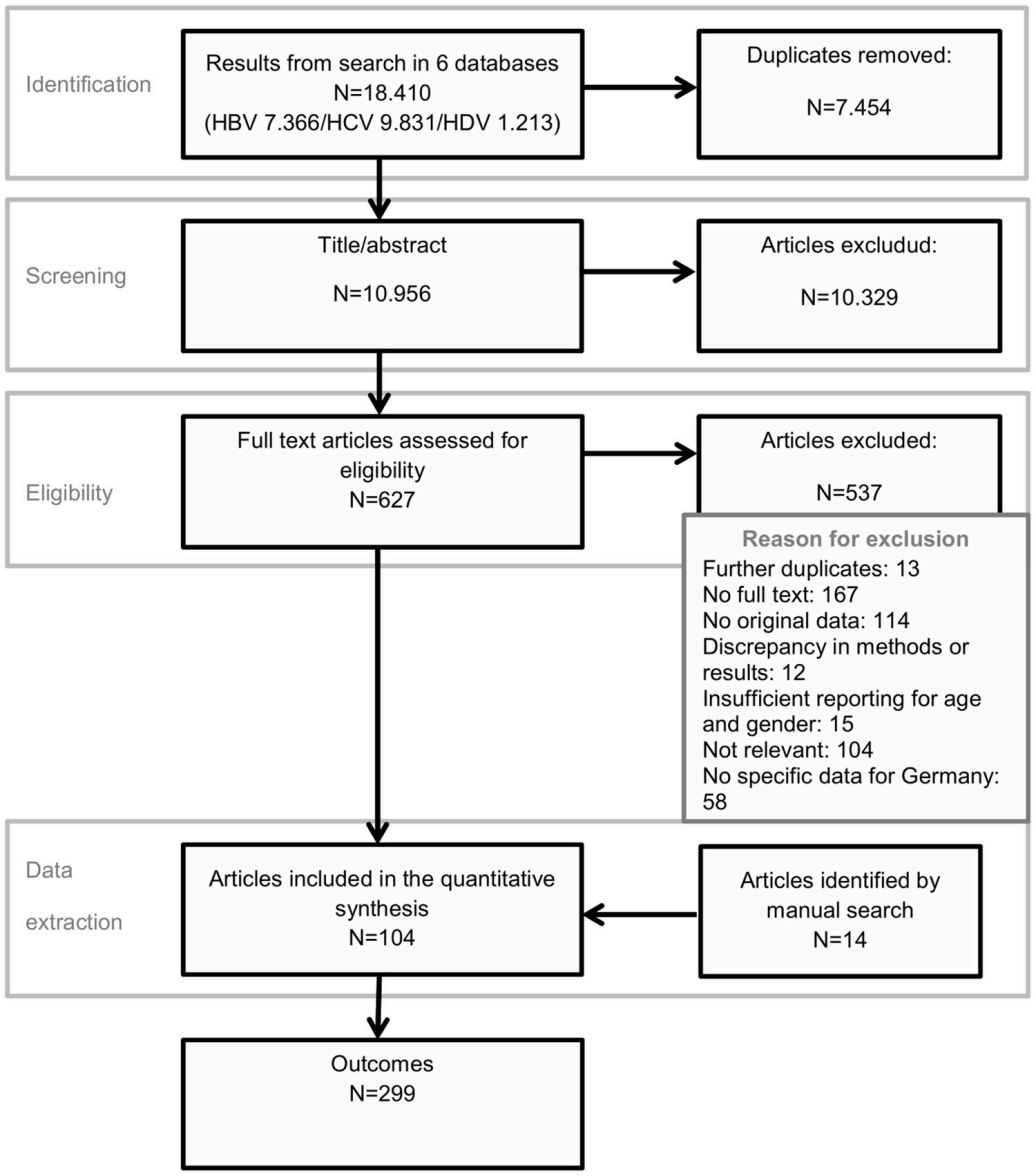
Flow chart of the study selection.

All publications were screened for relevance applying the study selection criteria The screening was conducted by two independent reviewers working in parallel, first for title and abstract followed by the full text. After the screening of 50 references, a validation of the screening process was conducted by comparing the screening results and discussing them within the team. Throughout the screening process, discrepancies were discussed and a third reviewer was consulted if consensus was not reached. The reasons for exclusion were documented only for full text publications (Fig 1), and track of the excluded studies was kept in a separate table (available upon request).

### Data extraction

**Fig 2:**
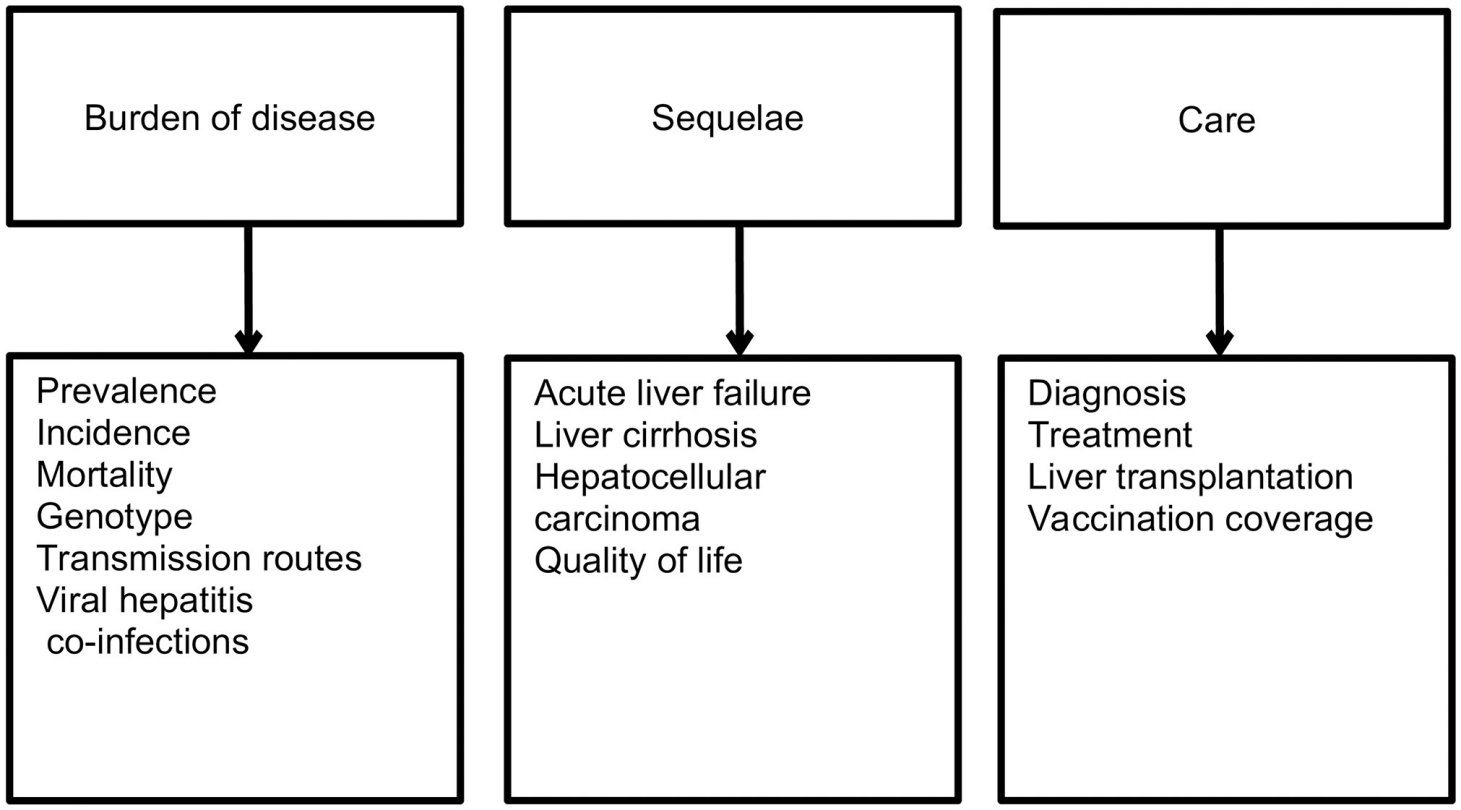
Outcome categories and sub-categories for the matrices.

Three main outcome categories (15 sub-categories) were derived from the research questions (outcome categories, Fig 2) to perform the extraction of the relevant data on outcome level. A standardised extraction sheet (Microsoft Excel) was developed containing 114 variables on outcome identifier, name and year of publication, author, study aim, study design (study population, study region, data collection period, sample, recruitment and data collection), outcome category and outcome results. After piloting the extraction sheet within the review team, relevant outcomes from eligible publications were extracted by one reviewer and cross-checked by a second one. Discrepancies between the reviewers were solved as described in the study selection process. The results were compiled in the extraction sheets. All extracted information was transferred to the sheet as stated in the corresponding publication. Minor errors in reporting/analysis (e.g. minor calculation errors) were discussed between the reviewers and corrected. For some publications more than one outcome was extracted. For each publication, the design of the corresponding study was determined. When more than one study design was used for different outcomes, the design with the highest evidence was reported.

### Data analysis

The identified literature was analysed for the extent of evidence, measured by the number of publications. Moreover sample size, place of data collection, study design, data collection tools, outcome category and population group were assessed on outcome and publication level. For this, the extraction sheets were subsequently collected and converted into a spreadsheet table by outcome. Relevant population groups were identified based on the WHO guidelines on Hepatitis B and C testing [3] and adapted to the German context. They included: i) the GP, ii) sub-populations which may represent a large proportion of the national population and therefore act as a proxy for the GP (blood donors, pregnant women and individuals born between 1946 and 1964, called baby boomers), iii) clinical populations (populations with non-VH related underlying disease and people with VH in hepatological care (PLWVH)), iiii) populations at risk for VH due to risk behaviour/exposure (household contacts of PLWVH, health-care workers (HCW), people living with HIV (PLWH), men who have sex with men (MSM), people in prison and closed settings, people who inject drugs (PWID), sex workers) or because they are part of a population with high VH seroprevalence, e.g. mobile or migrant populations from intermediate- and high-endemic countries. The study population of each outcome was assigned to a population group according to the definition used in the corresponding publication. When no definition was available, the review team discussed and allocated the outcomes of the publications. In general, only one population group was assigned per outcome, but exceptionally studies with outcomes in HIV positive MSM [16–20] were allocated to both population groups.

The outcomes from the spreadsheet table were classified in an evidence map by outcome category (Fig 2) and population group separately for HBV, HCV and HDV. In order to facilitate the readability, the outcomes in one cell were summarised on publication level (with number of outcomes still disclosed). Some references were assigned to more than one cell because they covered outcomes in different outcome categories/population groups. The evidence map contained information on place of data collection (local = one city/regional = more than one city in one region/nationwide = more than one city all over the country), size of study sample and quality grading on outcome level (see below).

The number of identified publications by outcome/population groups is not always corresponding to the number of studies conducted, as some outcomes of studies were split to more than one publication. In this work, we always referred to the number of outcomes and the number of publications, not to the number of studies conducted.

### Study quality

We developed a basic quality appraisal approach for of each outcome which included two criteria: national representation of the target population (regional or nationwide data collection) and adequacy of the study instrument for data collection. For details of the classification see Table 1. These criteria were selected due to their importance for viral hepatitis in Germany (i.e. are the results on national level and thereby representative for the national German population), and the study instrument to evaluate the potential risk of bias in the collected data. The total score for each criteria classified the outcomes in lower quality (0 and 1) and higher quality (2 and 3). Publications containing an outcome assessed with a quality index lower than 50% of the maximum were highlighted in the corresponding cell of the evidence map.

**Table 1:** Items for quality appraisal and classification.

Item for quality appraisal	0	1
**Representation of target population**	data collection regional	data collection nationwide
**Instrument for outcome measurement**	participant reported	biological marker clinical diagnosis/histology medical record physician reported
**Instrument for information on study population**	no information available	participant reported medical record report of physician

## Results

In total, the literature search in the six electronic databases retrieved 18,410 references for HBV, HCV and HDV. After removal of duplicates, 10,956 references were screened based on title and abstract. After removing irrelevant references, 627 full text publications were reviewed. Of these, 294 were excluded due to formal reasons (duplicates, no full text available, no original data), 27 due to poor quality of reporting and 187 due to content (data outside the time frame, not relevant for Germany or not relevant for the study question) and 29 due to other reasons. Ninety articles were included for data extraction and additional 14 references were identified by manual search. In total, 104 articles were included for data extraction [6, 7, 16–117]. Fifty-eight publications (83%) answered more than one research question and in total the 104 publications covered 299 outcomes. The stepwise selection of the included publications is shown in Fig 1.

Ninety-six (92%) of the publications were peer-reviewed journal articles, one a non-peer-reviewed article, one a book chapter and seven dissertations. Fifty-three (51%) of all identified publications were published in German. Eleven (11%) publications were studies with an overall study population smaller than 200 individuals and 51 (49%) with a study population larger than 1,000 individuals. On outcome level, for 60 outcomes (20%) less than 200 individuals were studied. In 40 publications (38%) the recruitment of the study population was nationwide. Seventy publications (67%) described studies with a cross-sectional study design, 27 (26%) with a cohort design, five (5%) contained surveillance data and two (2%) were on case-control studies. Eleven of the cross-sectional studies used data from the national school entry examinations and two publications with a cohort design were analyses of health insurance data. Case series analyses were always part of cohort studies. In 21 publications (20%), the outcomes on VH infection status, HBV vaccination status and/or VH treatment history were self-reported. In three publications, no information was available on how demographics of the study population were collected [38, 66, 107]. For the study characteristics of all included publications see S2 Table.

### Hepatitis B

Overall, 167 outcomes related to HBV were extracted from 83 publications. Six of them (in two publications) were in PLWH. One covered HBV/HCV co-infections in PLWH.

For the outcomes of 32 publications (39%), the data was collected nationwide, for the outcomes of 12 (14%) regional and for the outcomes of 38 (46%) locally. For one outcome (<1%) the regional representation of data colletion was not described. For 29 outcomes (in 16 publications) less than 200 individuals were observed. For 32 outcomes (in 20 publications) the outcome was self-reported (excl. four outcomes on knowledge about HBV-status and quality of life). For four outcomes (in three publications) information on how data was collected from the study population was not provided. Overall, the quality appraisal graded 8% of the outcomes (n = 14 in 11 publications) as lower quality (criteria see above).

Eighty HBV-related outcomes (in 47 publications) were classified as belonging to the category *burden of disease*, 25 (19) to the category *sequelae* and 62 (53) to the category *care*.

In 33 of all HBV related publications (40%) the study population was recruited from the GP or a proxy population, such as blood donors (seven publications), pregnant women (three publications) and baby boomers (one publication). For 12 publications (14%) different clinical populations were studied and 15 publications (18%) were on outcomes in PLWVH. Regarding populations at risk, in 14 of the publications (17%) the study population were HCW. Only three publications (4%) were about HBV outcomes in migrant/mobile populations and PWID each and one in MSM. One publication was about outcomes in PLWH and one studied HIV-positive MSM.

Considerable evidence was available for HBV prevalence in the GP and proxy populations, PLWVH and clinical populations, HCW, migrant/mobile populations and PWID; for HBV incidence in proxy populations for the GP; for transmission routes in the GP, PLWVH; for liver cirrhosis in PLWVH; for HCC in PLWVH and clinical populations; for proportion diagnosed in the GP; for proportion treated in PLWVH and for vaccination coverage in the GP, clinical populations, HCW and PWID.

Rare evidence was available for HBV prevalence and incidence in PLWH and MSM, for mortality and genotype distribution in PLWVH; for liver cirrhosis in the GP and clinical populations; for quality of life in PLWVH; for proportion of diagnosed in clinical populations; for proportion of treated in the GP and for vaccination coverage in household contacts of PLWVH, PLWH, MSM and migrant populations.

No evidence was available for HBV prevalence in household contacts of PLWVH; for HBV incidence in the GP, PLWVH and clinical populations, HCW, people in prison, migrant populations and PWID; for mortality in the GP and proxy populations, clinical populations and all populations at risk; for genotype distribution in the GP and proxy populations, clinical populations and all populations at risk, for transmission routes in proxy populations for the GP, in clinical populations and all populations at risk, for HIV co-infection in proxy populations for the GP, household contacts of PLWVH, people in prison and migrant/mobile populations; for acute live failure in the GP and proxy populations, PLWVH and all populations at risk; for liver cirrhosis in proxy populations for the GP and all populations at risk, for HCC in the GP and proxy populations and all populations at risk; for quality of life in all populations except PLWVH, for proportion diagnosed in proxy populations for the GP, PLWVH and all populations at risk; for proportion treated in proxy populations for the GP, clinical populations and all populations at risk; for liver transplantation in the GP and proxy populations, clinical populations and all populations at risk, and for vaccination coverage in proxy populations for the GP and people in prison.

The detailed extent of evidence for HBV in Germany is also shown in Tables 2 and 5.

**Table 2:** Matrix for Hepatitis B (publications by population and outcome category), publications. Shown are name_year of publication, sample size, place of data collection (local/regional (R) = one or more than one city in one region, nationwide (N) = more than one city all over the country).

	Burden of disease					Sequelae				Care				N studies
	Prevalence	Incidence	Mortality	Genotype	Transmission routes	Acute liver failure	Liver cirrhosis	Hepato-cellular carcinoma	Quality of life	Diagnosis	Treatment	Liver transplantation	Vacinnation coverage	
**General population**	[25], n = 1,0215, R[7], n = 13,065, N[32], n = 19,42/17, R (2) [50], n = 6,011, R [76], n = 7,047, N [110], n = 275, R [114], n = 1,373, R [115], n = 21,008, R				[7], n = 13,065, N [115], n = 21,008, R [50], n = 35, R		[50], n = 35, R			[50], n = 35, R [114], n = 1,373, R [115], n = 21,008, R	[50], n = 23, R		[7], n = 13065, N [32], n = 1,290, R** [46], n = 1,795, N [60], n = 1,468, R [75], n = 16,460, N [76], n = 7,047, N [6], n = 7,376, N [80], n = 803,875, N [81], n = 784,232, N [82], n = 755,997, N [83], n = 730,448, N [84], n = 732,187, N [85], n = 677,237, N [86], n = 694,236, N [87], n = 675,528, N [88]; n = 688,312, N [89], n = 700,146, N [90], n = 692,072, N	**23**
**Baby boomer**	[50], n = 1,235, R													**1**
**Blood donors**	[71], n = 92,660, N (2) [73], n = 548,608, N [113], n = 2,853,531 [111], n = 36,139, N [72], n = 2,948,018/ 3,116,643/ 3,074196, N (3) [104], n = 1,375,000, R [117], n = 3,694, R	[73], n = 548,608, N [113], n = 2,853,531, N [72], n = 2,948,018/ 3,116,643/ 3,074196, N (3)												**6**
**Pregnant women**	[21], n = 15,873, R [53], n = 5,518, R [54], n = 8,193, R													**3**
**People living with viral hepatitis**	[33], n = 218, R [41], n = 1,535, N [48], n = 10,326, N [51], n = 1,208, R [57], n = 327, R [58], n = 995, N [65], n = 250, R [66], n = 608, R [67], n = 79/151, N [112], n = 718, R [116], n = 148, N		[33], n = 35, R [66], n = 608, R	[27], n = 276, R	[41], n = 1,535, N [67], n = 255, N [66], n = 422, R [116], n = 160, N		[33], n = 218, R [41], n = 1,535, N [57], n = 128, R [65], n = 145, R [67], n = 140, N [66], n = 608, R [116], n = 160, N	[33], n = 218, R [38], n = 330, R** [67], n = 255, N [66], n = 608, R [106], n = 1,066, R [116], n = 160, N	[55], n = 201, R [67], n = 255, N		[33], n = 218, R [41], n = 1,535, N [55], n = 201, R** [57], n = 327, R [65], n = 250, R [67], n = 255, N [66], n = 608, R [116], n = 160, N	[33], n = 35, R [66], n = 608, R [116], n = 160, N		**15**
**Clinical populations**	[23], n = 417/417, R (2) [39], n = 313, R [40], n = 1,338, R [94], n = 463, R					[29], n = 134, R [43], n = 109, N	[96], n = 525, R [97], n = 236, R	[42], n = 484, R [52], n = 458, R [69], n = 1,1083/25, R (2) [74], n = 405, R		[32], n = 7, R			[39], n = 803, R [40], n = 1,338, R [94], n = 463, R	**13**
**Household contacts of People living with viral hepatitis**	[34], n = 384, R												[34], n = 384, R [55], n = 201, R (2)**	**2**
**Health care workers**	[22], n = 831, R [30], n = 367, R [108], n = 223, R [107], n = 366, R**												[22], n = 3,812, R [26], n = 82, R [30], n = 367, R [39], n = 28, R [56], n = 420, R** [78], n = 275, R** [81], n = 547, N [93], n = 242, R [101], n = 1,813, R [102], n = 168, R** [103], n = 432, R** [108], n = 223, R [107], n = 366, R** [109], n = 377, R**	**14**
**People living with HIV**	[19], n = 1838, N* [91], n = 918, R	[19], n = 1,838, N*											[19], n = 1,838, N* [91], n = 842, R	**2**
**People in prisons**														**0**
**Men who have sex with Men**	[19], (PLWH), n = 1,838, N*	[19], (PLWH) n = 1,838, N*											[19], (PLWH), n = 1,838, N* [18], n = 4,385, N	**2**
**Migrant/mobile populations**	[44], n = 793, R [45], n = 1,298, R [61], n = 488, R												[45], n = 795, R	**3**
**People who inject drugs**	[28], n = 2,077, R (2) [62], n = 420/404, R, (2) [63], n = 146, R												[28], n = 2,077, R [62], n = 430/404, R (2) [63], n = 146, R**	**3**
**N studies (N outcomes)**	**46 (55)**	**4 (6)**	**2 (2)**	**1 (1)**	**7 (7)**	**2 (2)**	**10 (10)**	**10 (11)**	**2 (2)**	**4 (4)**	**9 (9)**	**3**	**43 (46)**	

*outcome grouped in more than one population group

** outcome assessed with a quality index lower than 50% of the maximum

### Hepatitis C

In total, 146 outcomes in 60 identified publications were related to HCV. Ten of them (in five publications) were in PLWH.

For the outcomes of 25 publications (42%) the data collection was nationwide, for the outcomes of 10 (17%) regional and for the outcomes of 25 (42%) local. Less than 200 individuals were observed for 28 outcomes (in 14 publications). Overall, the quality appraisal graded 5% of the outcomes (n = 3 in 3 publications) as lower quality (criteria see above).

Ninety-eight HCV-related outcomes (in 50 publications) were assigned to the outcome category *burden of disease*, 24(19) to *sequelae* and 24 (19) to *care*.

Regarding populations, 12 publications (20%) focussed on the GP, added by one publication on baby boomers and one in pregnant women. In 28 publications (47%) clinical populations were studied. Publications on HCV-related outcomes in other populations at risk for VH were rare: five publications on studies in MSM, three in HCW, three in PWID, two in migrant/mobile populations and one in people in prison were identified. In six publications more than one population group was studied. For example, Kant et al. investigated the GP and additionally baby boomers as a sub-population [50]. Lugehetmann et al. studied PLWVH and the household contacts of PLWVH [55].

Considerable evidence was available for HCV prevalence in the GP (incl. proxy populations), HCW, PLWH and PWID; for HCV incidence in proxy population for the GP; for genotype distribution in PLWVH; for transmission routes in the GP, PLWVH and PLWH; for liver cirrhosis and HCC in PLWVH and clinical populations; for proportion diagnosed in the GP; for proportion diagnosed in the GP and for proportion treated in the GP and PLWVH.

Evidence was rare for HCV prevalence in clinical populations, people in prison, MSM and migrant/mobile populations; for HCV incidence in the GP, PLWH and MSM; for mortality in PLWVH; for genotype distribution in the GP, PLWH, MSM and PWID; for transmission routes in clinical populations, PLWH and MSM; for acute liver failure in PLWVH and clinical populations; for liver cirrhosis in the GP; for quality of life in PLWVH; for proportion of treated in clinical populations, PLWH, people in prison, MSM and PWID and for liver transplantation in PLWVH.

No evidence was available for HCV prevalence in pregnant women and household contacts of PLWVH; for HCV incidence in PLWVH and clinical populations, HCW, people in prison, migrant/mobile populations and PWID; for mortality in the GP and proxy populations, clinical populations and all populations at risk; for genotype distribution in proxy populations for the GP, clinical populations, household contacts of PLWVH, HCW, people in prison and migrant/mobile populations; for transmission routes in proxy populations for the GP, in household contacts of PLWVH, HCW, people in prison, migrant/mobile populations and PWID; for acute live failure in the GP and proxy populations and all populations at risk; for liver cirrhosis in proxy populations for the GP and all populations at risk; for HCC in the GP and proxy populations and all populations at risk; for quality of life in all populations except PLWVH; for proportion of diagnosed in proxy populations for the GP, PLWVH and clinical populations, and all populations at risk; for proportion of treated in proxy populations for the GP, household contacts of PLWVH, HCW and migrant/mobile populations and for liver transplantation in the GP and proxy populations, clinical populations and all populations at risk.

The extent of the evidence for HCV in Germany is shown in Tables 3 and 5.

**Table 3:** Matrix for Hepatitis C (publications by population and outcome category). Shown are name_year of publication, sample size, place of data collection (local/regional (R) = one or more than one city in one region, nationwide (N) = more than one city allover the country).

	Burden of disease					Sequelae				Care			N studies
	Prevalence	Incidence	Mortality	Genotype	Transmission routes	Acute liver failure	Liver cirrhosis	Hepatocellular carcinoma	Quality of life	Diagnosis	Treatment	Liver transplantation	
**General population**	[25], n = 1,0215/276, R (2) [32], n = 1,942, R [31], n = 15,070, N [36],n = 8,435, R [50], n = 6,011, R [6], n = 7,047, N [98], n = 3,000,000/10,379,N (2) [99], n = 5,464,191, N [100], n = 28,809, R [110], n = 275, R [114], n = 1,373,R [115], -n = 21,008, R	[98], n = 3,200,000, N		[25], n = 23, R [100], n = 320, R	[50], n = 56, R [100], n = 535, R [115], n = 21,008, R		[50], n = 56, R			[32], n = 6, R [50], n = 56, R [99], n = 5,464,191, N [100], n = 632, R [114], n = 1373, R [115], n = 21,008, R	[50], n = 48, R [99], n = 5,464,191, N [100], n = 535, R		**12**
**Baby boomer**	[50], n = 1,235, R												**1**
**Blood donors**	[71], n = 92,660, N (2) [73], n = 548,608, N [113], n = 2,853,531, N [111], n = 36,139, N [72], n = 2,948,018/ 3,116,643/ 3,074, N (3)	[73], n = 548,608, N [113], n = 2,853,531, N [72], n = 2,948,018/ 3,116,643/ 3,074196, N (3)											**5**
**Pregnant women**													**0**
**People living with viral hepatitis**	[41], n = 1,535, N [57], n = 327, R [67], n = 255, N		[105], n = 1,444, N [112], n = 718, R	[24], n = 172, R [35], n = 259, N [47], n = 1,440, N [51], n = 8,332, R [59], n = 23,893, N [68], n = 13,999, N [70] (PLWVH), n = 319, R* [77], n = 779, R [105], n = 1,444, N	[24], n = 172, R [35], n = 259, N [48], n = 10,326, N [47], n = 1,471, N [59], n = 13,422, N [70] (PLWVH), n = 319, R* [77], n = 779, R [105], n = 1,444, N	[35], n = 259, N [105], n = 1,444, N	[24], n = 53, R [48], n = 1,0326, N [47], n = 1,471, N [68], n = 11,435, N [77], n = 779, R [105], n = 1,444, N [112], n = 718, R	[38], n = 330, R** [105], n = 1,444, N [106], n = 1,066, R [112], n = 718, R	[48], n = 10,326,N		[24], n = 172, R [35], n = 259, N [47], n = 1,471, N [59], n = 23,893, N [68], n = 13,999, N [70] (PLWH), n = 319, R* [77], n = 779, R [105], n = 1,444, N [112], n = 718, R	[105], n = 1,444, N [112], n = 718, R	**18**
**Clinical populations**	[23], n = 417/417/407/ 398, R (4) [94], n = 463, R (2)				[94], n = 141, R	[29], n = 134, R	[64], n = 43, R [96], n = 525, R [97], n = 236, R	[42], n = 484, R [52], n = 458, R [69], n = 1,083/25, R (2) [74], n = 405, R			[94], n = 10, R**		**11**
**Household contacts of People living with viral hepatitis**													**0**
**Health care workers**	[22], n = 2,295, R [108], n = 223, R [107], n = 366, R**												**3**
**People living with HIV**	[91], n = 918, R [19], n = 1,838 [18], n = 874, N	[19], n = 1,838, R* [20], n = 95/73/59/11, R (4)		[19], n = 76, N* [70], n = 319, R*	[70], n = 319, R* [17], n = 101, N* [16], n = 38, R*						[70], n = 319, R* [16], n = 38, R*		**7**
**People in prisons**	[95], n = 14,187, N										[95], n = 1,4187, N		**1**
**Men who have sex with Men**	[19] (PLWH), n = 1,838, N* [18], n = 4,385##/874, N*	[20], (PLWH), n = 95/73/59/11; R (4)* [19] (PLWH), n = 1,838, R* [18], n = 4,385, N (2)		[19] (PLWH), n = 76, N*	[17], (PLWH) n = 101, N* [16], (PLWH) n = 38, R*						[16], (PLWH), n = 38, R*		**5**
**Migrant/mobile populations**	[45], n = 1,298, R [49], n = 236, R												**2**
**People who inject drugs**	[28], n = 1,361/516, R (2) [62], n = 420/404, R (2) [63], n = 146, R (2)			[28], n = 684, R							[28], n = 1,361, R		**3**
**N studies (N outcomes)**	**24 (46)**	**7 (12)**	**2 (2)**	**13 (13)**	**14 (14)**	**3**	**11(11)**	**8 (9)**	**1 (1)**	**6 (6)**	**16 (16)**	**2(2)**	

*outcome grouped in more than one population group

^##^not grouped in HIV because two diff outcomes with two denominators

** outcome assessed with a quality index lower than 50% of the maximum

### Hepatitis D

Seven-teen outcomes in eight publications were identified as HDV-related. One publication included nationwide data collected. The outcomes were classified as follows: seven (4) in burden of disease, eight (6) in sequelae, and two (1) in care. Coherent to the nature of HDV infection as a HBV superinfection, all identified publications analysed data collected in HBV positive clinical populations.

#### No publication was assessed as of low quality

Considerable evidence was available for HDV prevalence in PLWVH; rare evidence for mortality in PLWVH; transmission routes in PLWVH, HCV/HDV co-infections in PLWVH; liver cirrhosis in PLWVH; HCC in PLWVH and clinical populations; proportion of treated in PLWVH and liver transplantation in PLWVH. For all other combinations no evidence was available. For details of the evidence map, see Tables 4 and 5.

**Table 4:** Matrix for Hepatitis D (publications by population and outcome category). Shown are name_year of publication, sample size, place of data collection (local/regional (R) = one or more than one city in one region, nationwide (N) = more than one city all over the country).

	Burden of disease					Sequelae				Care			N studies
	Prevalence	Incidence	Mortality	Genotype	Transmission routes	Acute liver failure	Liver cirrhosis	Hepatocellular carcinoma	Quality of life	Diagnosis	Treatment	Liver transplantation	
**General population**													**0**
**Baby boomer**													**0**
**Blood donors**													**0**
**Pregnant women**													**0**
**People living with viral hepatitis**	[37], n = 1,307, R [41], n = 1,535, N [57], n = 327, R [79], n = 2,844, R		[37], n = 67, R		[37], n = 67, R		[37], n = 67, R	[37], n = 67/26, R (2)			[37], n = 67, R	[37], n = 67, R	**4**
**Clinical populations**						[29], n = 134, R		[42], n = 484, R [69], n = 1,083/25, R (2) [74], n = 405, R					**4**
**Household contacts of People living with viral hepatitis**													**0**
**Health care workers**													**0**
**People living with HIV**													**0**
**People in prisons**													**0**
**Men who have sex with Men**													**0**
**Migrant/mobile populations**													**0**
**People who inject drugs**													**0**
**N studies (N outcomes)**	**4(4)**	**0**	**1(1)**	**0**	**1(1)**	**1(1)**	**1(1)**	**4(6)**	**0**	**0**	**1(1)**	**1(1)**	

** outcome assessed with a quality index lower than 50% of the maximum

### VH co-infections

Fourteen publications included 16 outcomes regarding HBV/HCV co-infections and one publication reported one outcome regarding HCV/HDV co-infection. Considerable evidence was only available for HBV/HCV co-infections in PLWVH. Rare evidence was available for HBV/HCV co-infections in the GP and proxy populations, PLWH and PWID. For HCV/HDV co-infection, rare evidence was available in PLWVH.

For details of the evidence map, see Table 5.

**Table 5:** Matrix for publications on HBV/HCV and HCV/HDV co-infections (publications by population and outcome category). Shown are name_year of publication, sample size, place of data collection (local/regional (R) = one or more than one city in one region, nationwide (N) = more than one city allover the country).

	Co-infections		N studies
	HBV/HCV	HCV/HDV	
**General population**	[110], n = 275, R		**1**
**Baby boomer**			**0**
**Blood donors**	[113], n = 2,853,531, N [111], n = 36,139, N		**2**
**Pregnant women**			**0**
**People living with viral hepatitis**	[112], n = 718, R	[37], n = 67, R	**2**
**Clinical populations**	[32], n = 17, R [41], n = 1535, N [48], n = 10,326, N [51], n = 1,208, R [57], n = 327, R [58], n = 995, N [67], n = 255, N [94], n = 24, R		**9**
**Household contacts of People living with viral hepatitis**			**0**
**Health care workers**			
**People living with HIV**	[91], n = 918, R		**91**
**People in prisons**			**0**
**Men who have sex with Men**			**0**
**Migrant/mobile populations**			**0**
**People who inject drugs**	[28], n = 1361/516, R, (2)		**1**
**N studies (N outcomes)**	**12(13)**	**1(1)**	

** outcome assessed with a quality index lower than 50% of the maximum

## Discussion

The aim of our work was to give an extensive overview of the available evidence regarding the epidemiological situation of HBV, HCV and HDV in different population groups in Germany. We conducted a scoping review including a comprehensive literature search in electronic databases. We identified overall good evidence for HBV and HCV prevalence and HBV vaccination coverage in the GP and proxy populations. Evidence for these outcomes was weaker in populations at risk for VH. For HBV and HCV incidence and mortality, we identified large evidence gaps in all population groups. Outcomes on VH sequelae and care were mainly covered by data derived from case series of clinical populations with severe sequelae, treated in tertiary referral centres. For HDV the overall evidence available was scarce.

The methods of our scoping review were based on the PRISMA-ScR and the Cochrane collaboration (where applicable) to ensure the transparency of the process and reliability of this work. The review covered numerous important epidemiological topics leading to multiple study questions. Subsequently, we applied a broad search string to ensure that all literature with relevant outcomes were identified. We did not use search terms regarding study populations to not miss any relevant populations at risk for VH that we did not consider before. However, we used search terms and language restriction to narrow the search to articles about Germany. The restriction of the manual literature search to the screening of references during full text screening and published data from the Robert Koch Institute may have limited the completeness of the identified literature. However, we identified all our pre-defined index papers by the electronic search, which validates the sensitivity of our search strings. Relevant publications only detected by manual search were mainly articles published in national journals (e.g. the national surveillance journal) and not cited in electronic databases. To illustrate the identified body of literature, we defined outcome categories and population groups and, subsequently, created an evidence map. Limitations of using population groups may be (1) the challenge of allocating the study populations to the appropriate population group because of different naming and varying quality of the description of population characteristics in the corresponding publications and (2) the heterogeneity of the assigned study populations regarding age, sex and risk of VH infection. We defined proxy populations representing the GP (baby boomers, blood donors and pregnant women). These population groups still do not perfectly match with the GP, some populations at risk for VH (e.g. subpopulations of migrant/mobile people or PWID) may be under- or overrepresented in studies conducted in data coming from antenatal care but also in studies generally conducted in hospitals. In the blood donor surveillance individuals from high risk groups (e.g. PWID, sex workers) as well as individuals who are chronically infected with HIV, HBV or HCV and know their infection status are highly underrepresented as they are excluded from blood donation before screening if they report these risk factors in the pre-screening questionnaire. Therefore the prevalence of infection among those can be used only as the lower bound of prevalence in the general population. Apart from content-related variety of the outcomes and study populations, differences in study designs, methods and number of individuals observed among the included publications limited the comparability of evidence within and between different outcome categories and population groups. To provide a rough overview of the data quality for the evidence map we conducted a basic quality appraisal, assessing the national representation of the target population and the study instruments used to collect data regarding study population and outcome. The quality standard of the included publications according to the parameters assessed was overall good, though around one quarter of publications were of lower quality. However, we did not conduct a full critical appraisal according to the recommended criteria for systematic reviews (e.g. AMSTAR and others) for this broad scoping review and our quality appraisal does not reflect the overall quality of the studies included in our review. For further detailed analysis of the identified evidence, a comprehensive quality appraisal is essential to evaluate their risk of bias.

Regarding studies in the GP, data from the large population-based surveys DEGS1 (2008–2011) and KiGGS (Baseline study 2003–2006) provide solid evidence for adults and children, respectively, but are not very recent. The next examination study among children and young adults (KiGGS wave 2) has been carried out from 2014–2017 and a new population-based survey in adults is planned to be conducted by the RKI from 2020–2022. Studies in proxy populations included several large studies analysing test results of HBV screening during pregnancy and the national blood donor surveillance. Other evidence for the GP was mostly derived from large cross-sectional screening studies in hospital emergency departments or medical practices [25, 32, 36, 50, 100, 114, 115]. All these populations may represent the GP but it should be taken into account, that there might be a selection bias as population groups possibly differ in health care-seeking behaviour. Moreover sociodemographics and behaviour of treated patients might differ in different hospitals depending on the geographical location. In addition to the population-based surveys, we identified studies which had used statutory health insurance data for HCV analyses [98, 99]. This data covers 85% of the population, corresponding to the proportion of persons with statutory health insurance in Germany. This data may still be biased as some population groups in Germany do not have sufficient access to the German health system (due to legal or stigma-related barriers) and therefore may be underrepresented in health insurance based data. Analyses of data from the mandatory national school entry health examination provided valuable information on the HBV vaccination coverage in Germany. However, in adults (besides DEGS1) HBV vaccination in the GP was only covered by two large surveys on self-reported vaccination status [32, 46]. Regarding populations at risk, studies were more scarce, often smaller and more heterogenous regarding study population and outcome measurement tool. Data from HCW was often self-reported and derived from small sample sizes lacking robustness: except in two large studies on company doctors and medical students, respectively, [22, 101] the study populations of studies in HCW comprised less than 600 individuals. Studies in migrant/mobile populations varied in the target population. Two studies were conducted among refugees from different countries and minor refugees from Syria, respectively [44, 49, 61], while one large study was conducted in all patients with a migration background recruited in primary care regardless of country of origin, direct- or indirect migration and length of stay in Germany [45]. The risk for VH of the latter may be very different from the study populations of the other studies. Regarding MSM, most studies were conducted in PLWH. In the only survey recruiting MSM without a known HIV-infection, the VH infection and vaccination status was self-reported [18]. For PLWH on the other hand, one study in a non-MSM population recruited in HIV-care was identified [91]. People in prison were only addressed by one study analysing the results of HCV routine testing of prison inmates in Germany via questionnaires sent to the prison physicians [95]. For PWID, we identified only two larger studies [28, 62], one of which included serology data in PWID from several cities, while the other one only presented self-reported VH infection and vaccination status, resulting in a lower validity of the results [62].

Even if the overall evidence identified for HBV and HCV prevalence and HBV vaccination coverage was considerable, evidence for both outcomes is found insufficient in PLWH, MSM and HCV prevalence in mobile/migrant populations. Nonetheless, largest evidence gaps regarding burden of disease were identified for HBV and HCV incidence and mortality. While HCV incidence in the GP was calculated using the statutory health insurance data, for HBV incidence in the GP, evidence was only gained from the blood donor surveillance. No evidence was available in most populations at risk (HCW, migrant/mobile populations, people in prison and PWID). Moreover, evidence was rare in PLWH and MSM. For HBV and HCV mortality no evidence was available at all, except in clinical populations (PLWVH). Also for sequelae and care, evidence was scarce. Proportions of persons with VH related acute liver failure, hepatocellular carcinoma and liver transplantations were not covered by studies in the GP or defined populations at risk. Additionally, for HBV no evidence was available for liver cirrhosis, diagnosis and treatment in populations at risk. Evidence came mostly from PLWVH and clinical populations with (severe) sequelae and both populations may represent a mixed population of individuals at higher risk for VH than the GP. For HCV the evidence for treatment was slightly better with rare evidence available for PLWH, MSM, people in prison and PWID but not for HCW and migrant/mobile populations. However, the time frame of the literature search only covered the beginning of the new HCV treatment era, so an increasing amount of research regarding the proportion of HCV diagnosed and treated may be in the pipeline. Moreover, unpublished data at the time of the search may currently be available. All outcomes regarding sequelae and care were studied in PLWVH. Still, as mentioned above, parts of populations at risk for VH may not be sufficiently represented in clinical populations because of lacking access to health care. Therefore the burden of sequelae and proportion treated may be underestimated in these surveys.

One reason for the large evidence gaps regarding incidence studies might be the high costs for cohort recruiting and follow-up. This also applies to studies on mortality, sequelae and care. Here studies were mainly conducted in PLWVH, where recruitment is most convenient and cost-effective. Notification data could provide some information about notification incidence and time trends. Still, due to various reasons (e.g. underreporting and delay of notification), notification data does not reflect the real incidence. In addition, secondary data (e.g. from health insurance funds) could be a helpful and affordable source to gain more evidence regarding VH incidence, mortality, sequelae and care in the GP.

Regarding population groups, the largest evidence gaps were found for research in sex workers (not studied in any HBV- or HCV-related study), people in prison (only covered by a single study regarding HCV prevalence and HCV treatment) and household contacts of PLWVH. For studying populations at risk, using convenience samples may be necessary. Still, outreach work and innovative sampling methods (e.g. respondent driven sampling) should be applied to gain better access to some populations and to improve the representativeness of the target population.

In summary, generating evidence in all population groups is urgently needed regarding HBV and HCV incidence and mortality. Moreover, in addition to updating the evidence on prevalence from population-based surveys, focus should be targeted also on populations at risk. So far not sufficiently included subpopulations should be included in the study design or studies targeting these subpopulations must be carried out. This also applies to VH related sequelae and proportion treated. Knowing the care cascade in selected populations at risk is essential to evaluate the medical reachability of these groups, to plan targeted diagnostic and treatment programmes.

With our review we assessed the existing body of evidence regarding several epidemiological aspects of HBV, HCV and HDV in different population groups in Germany. The methods used enabled us to cover multiple research questions in one search to obtain a broad overview of all available evidence regarding VH epidemiology in Germany. We were also able to identify important research gaps regarding relevant outcomes and to overview the consideration of different population groups. Analyzing the identified data in detail, the review can be used as a reference to monitor the success of the national VH elimination strategy by comparing this baseline with periodic updates. Moreover, the identified evidence gaps can help to designate new targets for research in order to scale up operational research in fields with missing evidence to complete the picture and to step further towards VH elimination.

## Supporting information

S1 Table PRISMA-ScR checklist. (DOCX)

S2 Table Characteristics of the included studies. (XLSX)

S1 Text Search strategy. (DOCX)

S1 Fig In- and exclusion criteria. (DOCX)
