# Supplementary material for: The epidemiology of Hepatitis B, C and D in Germany: A scoping review
Source: PLoS One. 2020 Mar 9;15(3):e0229166. doi: 10.1371/journal.pone.0229166 (PMC7062254; doi:10.1371/journal.pone.0229166)
Supplement: S1 Text — (DOCX) [file pone.0229166.s003.docx]

**S1_Text: Search strategy**

1. **Search in Medline, EMBASE, Europe PMC, Scopus (full-text search; date of last search: 9 March 2017, search restricted to 1 January 2005 – 9 March 2017; filters: English and German language):**

#1 hepatitis b

#2 HBV

#3 hepatitis c

#4 HCV

#5 hepatitis d

#6 HDV

#7 viral hepatitis

#8 European Union

#9 EU

#10 EEA

#11 europ*

#12 german*

#13 deutsch*

#14 Berlin

#15 Hamburg

#16 Munich

#17 Cologne

#18 Frankfurt

#19 Stuttgart

#20 Dusseldorf

#21 Dresden

#22 Dortmund

#23 epidemiol*

#24 seroepidem*

#25 screen*

#26 prevalence*

#27 seroprevalence*

#28 incidence*

#29 “burden of disease”

#30 “disease burden”

#31 burden

#32 morbid*

#33 DALY*

#34 mortality*

#35 letal*

#36 risk factor*

#37 transmission

#38 infection risk*

#39 viraemic rate*

#40 infection rate*

#41 therapy rate*

#42 healing rate*

#43 reinfection*

#44 vaccination rate*

#45 vaccination coverage

#46 modelling

#46 epidemiology

#47 #1 OR #2 #7

#48 #3 OR #4 OR #7

#49 #5 OR #6 OR #7

#50 #8 OR #9 OR #10 OR #11 OR #12 OR #13 OR #14 OR #15 OR #16 OR #17 OR #18 OR #19 OR #20 OR #21 OR #22

#51 #23 OR #24 OR #25 OR #26 OR #27 OR #28 OR #29 OR #30 OR #31 OR #32 OR #33 OR #34 OR #35 OR #36 OR #37 OR #38 OR #39 OR #40 OR #41 OR #42 OR #43 OR #44 OR #45 OR #46

#52 #23 OR #24 OR #25 OR #26 OR #27 OR #28 OR #29 OR #30 OR #31 OR #32 OR #33 OR #34 OR #35 OR #36 OR #37 OR #38 OR #39 OR #40 OR #41 OR #42 OR #43 OR #46

#53 #47 AND #50 AND #51

#54 #48 AND #50 AND #52

#55 #49 AND #50 AND #51

1. **Search in CC Med (date of last search: 9 March 2017, search restricted to 2005 – 2017; filters: German language):**

#1 hepatitis b

#2 HBV

#3 hepatitis c

#4 HCV

#5 hepatitis d

#6 HDV

#7 virale hepatitis

#8 epidemiolog*

#9 seroepidemiolog*

#10 prävalenz

#11 seroprävalenz

#12 inzidenz

#13 mortalität

#14 morbidität

#14 krankheitslast

#15 folgeerkrankung*

#16 letal

#17 übertragung

#18 diagnoserate

#19 therapierate

#20 infektion

#21 impf*

#22 #1 OR #2 OR #7

#23 #3 OR #4 OR #7

#24 #5 OR #6 OR #7

#25 #8 OR #9 OR #10 OR #11 OR #12 OR #13 OR #14 OR #15 OR #16 OR #17 OR #18 OR #19 OR #20 OR #21

#26 #8 OR #9 OR #10 OR #11 OR #12 OR #13 OR #14 OR #15 OR #16 OR #17 OR #18 OR #19 OR #20

#27 #22 AND #25

#28 #23 AND #26

#29 #24 AND #25

1. ***Search in Base Bielefeld (date of last search: 9 March 2017, search restricted to 2005 – 2017; filters: German language):***

#1 Hepatitis B

#2 HBV

#3 Hepatitis C

#4 HCV

#5 Hepatitis D

#6 HDV

#7 virale Hepatitis

#8 deutsch*

#9 germ*

#10 #1 OR #2 OR #7

#11 #3 OR #4 OR #7

#12 #5 OR #6 OR #7

#13 #8 AND #9

#14 #10 AND #13

#15 #11 AND #13

#16 #12 AND #13
