# Supplementary material for: The epidemiology of Hepatitis B, C and D in Germany: A scoping review
Source: PLoS One. 2020 Mar 9;15(3):e0229166. doi: 10.1371/journal.pone.0229166 (PMC7062254; doi:10.1371/journal.pone.0229166)
Supplement: S1 Fig — (DOCX) [file pone.0229166.s004.docx]

| **Inclusion criteria** | **Exclusion criteria** |
| --- | --- |
| 1. **Type of publication:**    - 1. **publications:** only if full text available   Peer-reviewed article, non-Peer-reviewed article, report, dissertation   - - 1. **non-published data**   published and non-published data without further explanation only after consulting the data provider (e.g. secondary data, registry data)   1. **Type of work:** only original work with a clear origin of the analysed data 2. **Type of data:** original data (observational studies, e.g. cross-sectional studies, cohort studies, cases of case-control studies, case series; surveillance data), reviews with a systematic approach/evidence based guidelines with a underlying systematic review, secondary/registry data 3. **Quality standards:** report of n study population, age groups and sex study population, place of data collection 4. **Time frame**   data collection not finished before 1/1/2005 published after 1/1/2005   1. **Content:**    1. Relation to:  - Hepatitis B, C und D - Germany - Humans   1. Epidemiological relevance:   distribution, prevlance, incidence, mortality, morbidity related to   - - 1. Hepatitis B, C und D infection (     2. sequelae (only acute liver failure, hepatocellular carcinoma, liver cirrhosis)     3. co-Infection: only HIV and HBV/HCV/HD     4. distribution of subtypes     5. transmission routes     6. quality of life   1. Data related to:      1. **diagnosis rate** (proportion of unknown/known infections )      2. **therapy rate** (indicated, started, finished, interrupted)      3. **vaccination coverage (complete, incomplete)**      4. **transplantation rate** directly related to Hepatitis B, C, D | 1. **Type of publication:**    1. **publications**:   **No** full text existing or available for study team   - 1. **Non-published data**   **No** consultation of the data provider possible   - 1. **Data already used in other publication:**   Publication without further information regarding the study questions   1. **Type of work:** *no* original work   e.g. expert opinions, narrative reviews, editorials, comments   1. **Type of data:** e.g. method papers (containing no data), single case reports, RCT 2. **Quality standards:** No report of: n study population, age groups and sex study population, place of data collection 3. **Time frame:** data collection finished before 1/1/2005 published before 1/1/2005 4. **Publications reporting identical outcomes of the same study and no new and relevant aspects according to the research questions (newer publication excluded)** 5. **Content:**    1. No relation to :       1. Hepatitis B, C und D       2. Germany       3. Humans    2. No relevance for study question:       1. other topics than in study questions       2. pathogen: virology, clinical symptoms       3. other sequelae than acute liver failure, hepatocellular carcinoma, liver corrhosis       4. other co-infections than HIV and HBV/HCV/HDV    3. Data related to:       1. **diagnosis**: diagnostics, lab methods       2. **therapy**: single drugs, therapy scheme, therapy in immunocompromised patients, side effects       3. **vaccination**: vaccines, vaccination schedules, vaccination of immunocompromised, side effects       4. **liver transplantation** (LTX): risks, process, complications, drug therapy, outcome, re-transplantation, LTX under specific circumstances    4. Data related to:       1. prevention       2. level of care       3. costs       4. politics |
